# Comprehensive analysis of ferritinophagy-related genes and immune infiltration landscape in diabetic retinopathy

**DOI:** 10.3389/fendo.2023.1177488

**Published:** 2023-07-14

**Authors:** Fenfen Yu, Congyao Wang, Yihua Su, Tingting Chen, Wenhui Zhu, Xia Dong, Wanyi Ke, Leqi Cai, Shasha Yang, Pengxia Wan

**Affiliations:** ^1^ Department of Ophthalmology, The First Affiliated Hospital, Sun Yat-sen University, Guangzhou, China; ^2^ Department of Ophthalmology, Guangzhou First People’s Hospital, The Second Affiliated Hospital, South China University of Technology, Guangzhou, China

**Keywords:** diabetic retinopathy, ferritinophagy, immune landscape, differentially expressed genes, BECN1, HERC2, ATG7, BCAT2

## Abstract

**Background:**

Diabetic retinopathy (DR) is deemed a microangiopathy and neurodegenerative disorder, which is a primary reason of visual impairment in the world. Ferritinophagy is a critical regulator of ferroptosis and has a vital part in the etiopathogenesis of DR. Nevertheless, its molecular mechanism in DR remains to be expounded.

**Methods:**

The GSE146615 dataset was adopted to identify ferritinophagy-related differentially expressed genes (FRDEGs). The interactions and biological functions of the genes were described by means of functional enrichment analysis (FEA). The enriched gene sets were analyzed utilizing gene set enrichment analysis (GSEA) and gene set variation analysis (GSVA). Identification of hub genes was performed utilizing protein–protein interaction (PPI) analysis. mRNA–miRNA, mRNA–transcription factors (TF), mRNA–drugs, mRNA–RNA-binding proteins (RBP) interaction networks were constructed. In addition, datasets GSE60436 and GSE94019 were utilized for validation. The diagnostic performance of FRDEGs was assessed by means of receiver-operating characteristic curve monofactor analysis, followed by immune infiltration analysis. Lastly, quantitative real-time polymerase chain reaction (qRT-PCR) was implemented to analyze the validation of genes.

**Results:**

In total, the identification of eight FRDEGs was completed utilizing differential expression analysis. FEA mainly implicated the autophagy of mitochondrion, mitochondrion disassembly, autophagosome assembly, and organization pathways. GSEA and GSVA mainly implicated the interferon alpha response, ultraviolet response up, interferon gamma response, apical junction, pical surface, and allograft rejection pathways. BECN1 and HERC2 displayed high diagnostic accuracies in validation sets. Immune infiltration analysis revealed that several immune cells related to ferritinophagy may be play potential roles in DR. Finally, qRT-PCR was utilized to validate the upregulated expression of BECN1 as well as the downregulated expression of BCAT2 and ATG7 in the DR model.

**Conclusion:**

BECN1, HERC2, ATG7, and BCAT2 act as potential biomarkers for DR and might regulate ferritinophagy and the immune microenvironment to influence its development and progression. This research can provide new insights into pathogenesis of DR related to ferritinophagy.

## Introduction

Diabetic retinopathy (DR) is a type of frequent diabetic complication as well as the main reason for visual loss among the working-age population globally (1, 2). In 2020, 103 million adults suffered from DR in the world, and the number is estimated to grow to 160 million by 2045 (3). A heavier social burden and increased healthcare costs could be expected. Numerous reports have pointed out that endoplasmic reticulum stress, oxidative stress, glutamate toxicity, autophagy, and apoptosis could trigger retinal neural dysfunction, retinal vascular degeneration, neovascularization, and retinal inflammation (4–7). Nevertheless, existing therapy is mainly used in advanced stages of DR, and the long-term effect is unsatisfactory (8). Hence, to improve clinical outcomes, there is a pressing need to identify novel biomarkers and treatment strategies for DR.

Ferritinophagy is a selective autophagic degradation of ferritin, which results in cytosolic iron overload in the shape of ferrous (Fe^2+^) and lipid peroxidation, ultimately causing ferroptosis and cell death (9–11). Evidence has depicted that ferritinophagy has a vital role in the development and progression of diabetic complications (12). The renal biopsy results of diabetic nephropathy have been reported to display higher transferrin expression and iron deposition in renal tubular epithelial cells than the healthy control group (13). Ferritinophagy regulation by suppression of DNA (cytosine-5)-methyltransferase 1 (DNMT-1) could decrease ferroptosis in diabetic myocardial I/R injury (14). Rev-Erbs agonist SR9009 relieves myocardial I/R injury by downregulating ferritinophagy/ferroptosis signaling in type-2 diabetic rats (15). Recent studies have shown that ferritinophagy may be involved in DR. High glucose-induced upregulation of thioredoxin-interacting protein (TXNIP) and associated redox stress trigger ferritinophagy in a human retinal pigment epithelial cell line (ARPE-19) (16). Nevertheless, the DR-related biomarkers, signaling pathways, and molecular regulatory mechanisms of ferritinophagy remain to be further explored.

We enrolled an original training dataset consisting of peripheral blood lymphoblastic cell line samples and two validation datasets consisting of intraocular fibrovascular membrane samples in our research. First, the Gene Expression Omnibus (GEO) database was adopted to collect RNA-sequencing datasets, and the GeneCards database was adopted to download ferritinophagy-related genes. In total, the identification of eight ferritinophagy-related differentially expressed genes (FRDEGs) was completed. The interactions and biological functions of the genes were described using functional and pathway enrichment analysis. The analysis of enriched gene sets was implemented by means of Gene Set Enrichment Analysis (GSEA) and Gene Set Variation Analysis (GSVA). Identification of hub genes was completed through protein–protein interaction (PPI) analysis. Following that, mRNA–miRNA, mRNA–drugs, mRNA–transcription factors (TF), mRNA–RNA-binding protein (RBP) interaction networks were constructed. In addition, two other GEO datasets were utilized for validation. The immune microenvironment in DR was analyzed by CIBERSORT. Finally, quantitative real-time polymerase chain reaction (qRT-PCR) was conducted to analyze and verify FRDEGs in the model of DR *in vitro*. The research can provide new insights into molecular pathogenesis, potential biomarkers and therapeutic targets for ferritinophagy in DR.

## Materials and methods

### Acquisition and download of dataset

The GEO database (http://www.ncbi.nlm.nih.gov/geo/) was accessed to acquire the mRNA expression profile datasets GSE146615, GSE60436, and GSE94019 from *Homo sapiens*. GSE146615 (as training set) includes 42 individuals without diabetes, 52 patients with DR, and 50 diabetic patients without retinopathy. Peripheral blood from 144 individuals was utilized to acquire lymphoblastoid cell lines, which were then treated with standard glucose and high glucose separately. The data of 52 DR and 42 normal samples were extracted for subsequent analysis in this study. As a validation set, GSE60436 included six fibrovascular membrane samples excised from DR patients and three normal human retina samples, GSE94019 contained nine fibrovascular membrane samples and four normal controls. The flow chart of data analysis is displayed in **Supplementary Figure S1**.

### Analysis of FRDEGs

The *Limma* package in R software was applied for analyzing differentially expressed genes (DEGs) in DR and normal control samples. DEGs were determined as the genes with *p*-values < 0.05 and |Log FC| > 0. GeneCards database (https://www.genecards.org/) was applied to screen 20 ferritinophagy-related genes in total. The Venn plots were employed to visualize the intersections of DEGs and ferritinophagy-related genes. A heat map was utilized to display expression profiles of FRDEGs in GSE146615 dataset.

### Functional enrichment analysis of FRDEGs

Gene Ontology (GO) analysis is a common approach for EFA of large-scale studies, comprising biological process (BP), molecular functions (MF), and cellular component (CC). Kyoto Encyclopedia of Genes and Genomes (KEGG) is a database resource including rich information on biological pathways, genomes, diseases, chemical substances, and medications. ClusterProfiler package in R software was employed for functional enrichment analysis (FEA) of FRDEGs, comprising the two methods mentioned above. The statistical difference was deemed to be both *p*-value and FDR (Q value) lower than 0.05, with Benjamini–Hochberg conducted for *p*-value correction.

### GSEA and GSVA of FRDEGs

The differential gene sets of DR samples and normal control samples in the GSE146615 dataset were explored by means of GSEA. Molecular Signatures Database (MSigDB) was employed to download h.all.v7.2.symbols.gmt (Hallmark) as a reference gene set. The seed was 2020, and the number of calculations was 10,000, with Benjamini–Hochberg conducted for *p*-value correction. The cutoff criteria were determined as statistical differences at FDR < 0.25 and *p* < 0.05. The top four results were screened and visualized according to the normalized enrichment score (NES).

Meanwhile, individual samples were assessed by means of GSVA through a nonparametric approach in GSE146615 dataset. MSigDB was employed to download h.all.v7.5.2.symbols.gmt (Hallmark) as a reference gene set. The statistical difference was deemed to be *p* < 0.05. Outcomes were shown by heat maps and group comparison plots.

### PPI analysis of FRDEGs

With the minimum required interaction score set at a medium confidence of 0.150, STRING database was adopted for PPI analysis of FRDEGs. The visualization and construction of PPI network were then implemented by means of the Cytoscape v 3.9.1 software.

### Interaction analysis of miRNA targets with FRDEGs

Starbase is a database for searching for candidate miRNA targets from Degradome-Seq and CLIP-Seq data that provides multiple visual interfaces for miRNA targets. miRNA–ncRNA, miRNA–mRNA, miRNA–RNA, and RNA–RNA data are included abundantly in the database. MiRDB is a database for miRNA functional annotation as well as miRNA target prediction. starBase and miRDB databases were applied to predict the microRNAs interacting with FRDEGs, and Cytoscape software was utilized to select the overlapped miRNA–mRNA pairs.

CHIPBase v2.0 (https://rna.sysu.edu.cn/chipbase/) is an integration of resource and platform that has been utilized to identify a great number of binding motif matrices and binding sites from ChIP-seq data of DNA-binding proteins as well as predict millions of transcriptional regulatory relationships between genes and TFs. HTFtarget database (http://bioinfo.life.hust.edu.cn/hTFtarget) is an integration of resource and platform for the regulation of human TFs and their targets. TFs binding to FRDEGs were screened from the CHIPBase database and the hTFtarget database.

The Comparative Toxicology Genomics Database (CTD) (http://ctdbase.org/) is a publicly accessible database containing manually curated resources on gene–disease, chemical–disease, chemical–gene, and chemical–protein interactions. The CTD database was utilized to predict the interaction of potential medicines or small-molecule compounds with FRDEGs.

In addition, the ENCORI database was used to predict RBPs interacting with FRDEGs. All interaction networks were visualized by the Cytoscape software v3.9.1.

### Receiver operating characteristic validation of FRDEGs

The diagnostic performance of FRDEGs in the GSE146615, GSE60436, and GSE94019 datasets was assessed by means of receiver operating characteristic (ROC) monofactor analysis. Hiplot software was applied to visualize ROC curve. Genes were believed to have diagnostic value when the area under the curve (AUC) of ROC was greater than 0.7.

### Immune cell infiltration-related analysis

The relative compositions of immune cells infiltrated into the samples in the GSE146615 dataset were assessed by means of the CIBERSORT algorithm. The LM22 signature matrix acted as a reference expression signature (1,000 permutations). In case of CIBERSORT *p* < 0.05, the data were screened and retained for the subsequent analysis. Thereafter, data with an immune cell enrichment score of > 0 were screened, and the specific outcomes of immune cell infiltration matrix were eventually acquired. R ggplot2 package was adopted for visualization of results from CIBERSORT.

### Cell culture and treatment

After supplementing with fetal bovine serum (10%) and antibiotic–antimycotic (1%) supplied by Gibco (Grand Island, New York, USA), DMEM/F12 medium was supplemented to ARPE-19 (Cell Bank, Chinese Academy of Sciences, Beijing, China) for cultivation with 5% CO_2_ at 37°C. After the cell density reached 80%, ARPE-19 was seeded in a 12-well plate (1.2 × 10^6^ cells/well). Afterwards, cells were distributed to a HG-treated (30 mM glucose) group and a normal group (cultivated in DMEM with a 5.5-mM basal concentration of glucose), with both groups receiving 48 h of culture.

### Quantitative real-time PCR

Trizol Reagent (Invitrogen, Waltham, Massachusetts, USA) was employed for total RNA extraction of ARPE-19 cells in accordance with the protocol. TransScript All-in-One SuperMix kits (Beijing TransGen Biotech, Beijing, China) were applied to obtain the cDNA by means of a reverse transcription assay. 2× SYBR Green qPCR Master Mix kits (Servicebio, Wuhan, China) were utilized to conduct QRT-PCR reactions in Eco™ Real-Time PCR System (illumina, San Diego, California, USA). The 2^−ΔΔCt^ method was applied for analysis of mRNA expression level, and β-actin was utilized for the normalization of relative gene mRNA expression. **Supplementary Table S1** displays the gene primers.

### Statistical method

R version 4.2.1 was applied for data processing and analysis. The differences between continuous variables with a normal distribution were examined with Student’s *t*-test. The differences between continuous variables without a normal distribution were examined with Mann–Whitney *U* test. Categorical variables were compared with Chi-square test or Fisher’s exact test. The statistical differences were deemed to *p*-values lower than 0.05.

GraphpadPrism software (version 3.6.2) was employed to statistically analyze experimental data. Each experiment was conducted for at least three repetitions with triplicate samples to acquire results. Two-tailed Student’s *t*-test was applied for the comparison of sample gene expression, with *p* < 0.05 representing statistical significance.

## Results

### Identification of FRDEGs

**Figure 1:**
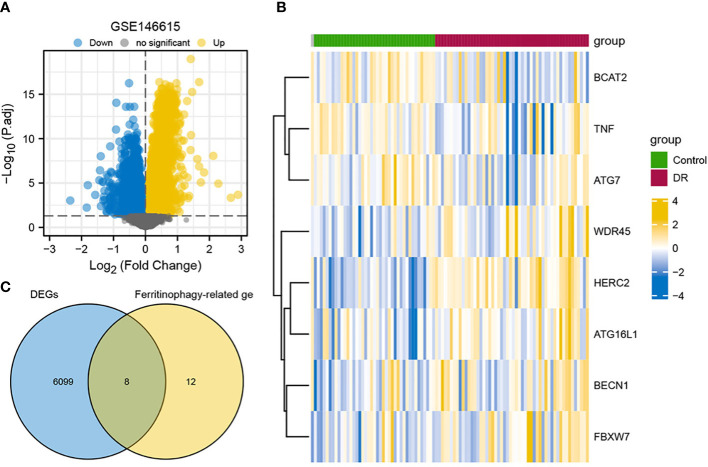
Overview of FRDEGs in two groups. **(A)** Volcano plot of DEGs between two groups in GSE146615 dataset, with blue nodes denoting downregulated genes, yellow nodes denoting upregulated genes, and gray nodes representing no statistical significance in contrast to controls. **(B)** Heat map of eight FRDEGs, with blue representing downregulation and yellow representing upregulation. **(C)** Intersection of DEGs in GSE146615 with ferritinophagy-related genes. DR, diabetic retinopathy; DEGs, differentially expressed genes; FRDEGs, ferritinophagy-related DEGs.

DEGs in lymphoblastoid cell line-derived RNA samples from the DR group and the control group were analyzed using the *Limma* package (**Supplementary Figures S2A, B**). There were 6,107 DEGs identified by screening, of which 2,696 were upregulated genes and 3,411 were downregulated genes (**Figure 1A**). Next, Venn diagram was applied to conduct and display the intersection of DEGs with ferritinophagy-related genes (**Figure 1B**). A total of eight FRDEGs were obtained, including HERC2, ATG16L1, WDR45, BCAT2, FBXW7, BECN1, TNF, and ATG7. In the DR group, the upregulated genes were WDR45, HERC2, ATG16L1, BECN1, and FBXW7. In the control group, the upregulated genes were BCAT2, TNF, and ATG7. The standardized expression of FRDEGs (five upregulations and three downregulations) was displayed by means of heat map (**Figure 1C**).

### Functional and pathway enrichment of FRDEGs

**Figure 2:**
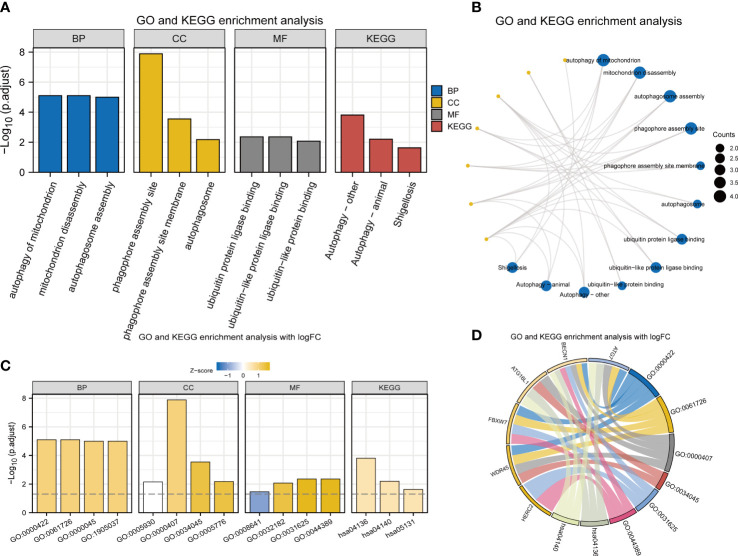
GO and KEGG enrichment analyses of 8 FRDEGs. **(A)** Bar graphs of GO and KEGG analyses. **(B)** Ring network diagram of GO and KEGG analyses. **(C)** Bar graphs of GO and KEGG analyses combined with logFC. **(D)** Chordal graph of GO and KEGG analyses combined with log FC. GO, Gene Ontology; FRDEGs, ferritinophagy-related differentially expressed genes; KEGG, Kyoto Encyclopedia of Genes and Genomes; BP, biological process; CC, cellular component; MF, molecular function.

BP, CC, and MF were included in GO annotations of FRDEGs. In the GO-BP analysis, the most prominent projects involved autophagy of mitochondrion, mitochondrion disassembly, and autophagosome assembly. In GO-CC analysis, the differential genes were significantly enriched in phagophore assembly site (PAS), PAS membrane, and autophagosome. The results of enrichment analysis in GO-MF were ubiquitin protein ligase binding, ubiquitin-like protein ligase binding, and ubiquitin-like protein binding. Autophagy-other, autophagy-animal, and Shigellosis were the main enriched pathways in KEGG analysis (**Table 1**; **Figures 2A, B**). Based on the results of enrichment analysis, the corresponding standard score (Z-score) of each pathway was calculated by combining the molecular logFC and plotted by the bar chart (**Figures 2C, D**).

**Table 1:** Results of GO and KEGG enrichment analyses.

Terms	Descriptions	*p*-values	adj. *p*-values	*q*-values
Biological processes				
GO:0000422	Autophagy of mitochondrion	1.75*e*−08	7.95*e*−06	3.03*e*−06
GO:0061726	Mitochondrion disassembly	1.75*e*−08	7.95*e*−06	3.03*e*−06
GO:0000045	Autophagosome assembly	3.98*e*−08	1.01*e*−05	3.84*e*−06
GO:1905037	Autophagosome organization	4.52*e*−08	1.01*e*−05	3.84*e*−06
GO:1903008	Organelle disassembly	5.55*e*−08	1.01*e*−05	3.84*e*−06
Cellular component				
GO:0000407	Phagophore assembly site	3.98*e*−10	1.31*e*−08	8.38*e*−09
GO:0034045	Phagophore assembly site membrane	1.72*e*−05	2.84*e*−04	1.81*e*−04
GO:0005776	Autophagosome	6.05*e*−04	0.007	0.004
GO:0005930	Axoneme	0.001	0.007	0.005
GO:0097014	Ciliary plasm	0.001	0.007	0.005
Molecular functions				
GO:0031625	Ubiquitin protein ligase binding	2.29*e*−04	0.004	0.002
GO:0044389	Ubiquitin-like protein ligase binding	2.74*e*−04	0.004	0.002
GO:0032182	Ubiquitin-like protein binding	7.98*e*−04	0.009	0.003
GO:0008641	Ubiquitin-like modifier activating enzyme activity	0.005	0.034	0.014
GO:0032183	SUMO binding	0.007	0.034	0.014
KEGG pathway				
hsa04136	Autophagy—other	1.96*e*−06	1.55*e*−04	1.17*e*−04
hsa04140	Autophagy—animal	1.59*e*−04	0.006	0.005
hsa05131	Shigellosis	8.92*e*−04	0.023	0.018

### GSEA and GSVA of FRDEGs

**Figure 3:**
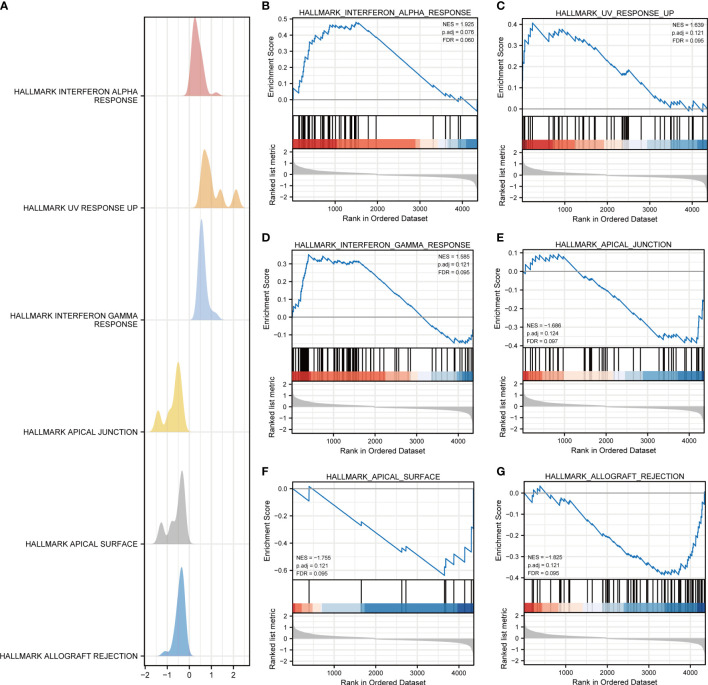
GSEA for dataset GSE146615. **(A)** Mountain map of GSEA results of differential genes (DEGs). Several pathways and biological processes were differentially enriched, including **(B)** HALLMARK_INTERFERON_ALPHA_RESPONSE, **(C)** HALLMARK_UV_RESPONSE_UP, **(D)** HALLMARK_INTERFERON_GAMMA_RESPONSE, **(E)** HALLMARK_APICAL_JUNCTION, **(F)** HALLMARK_APICAL_SURFACE, and **(G)** HALLMARK_ALLOGRAFT_REJECTION. NES, normalized enrichment scores; FDR, false-discovery rate.

With the aim of further expounding the various pathways involved in DR, GSEA was implemented in DR and control groups in the dataset GSE146615. An unbiased global search for genes that coordinated regulation in predefined gene stets was implemented to assess the microarray data (**Table 2**; **Figure 3A**). The analysis revealed the significant enrichment of genes in six pathways, including interferon alpha (INF-α) response (**Figure 3B**), ultraviolet (UV) response up (**Figure 3C**), interferon gamma (INF-γ) response (**Figure 3D**), apical junction (**Figure 3E**), pical surface (**Figure 3F**), and allograft rejection (**Figure 3G**).

**Table 2:** GSEA results.

ID	Enrichment score	Normalized enrichment scores	*p*-value	*q*-value
HALLMARK_INTERFERON_ALPHA_RESPONSE	0.4798755	1.925361718	0.001618123	0.059615057
HALLMARK_ALLOGRAFT_REJECTION	−0.386761109	−1.825360745	0.007915567	0.094618569
HALLMARK_APICAL_SURFACE	−0.637182006	−1.754959124	0.011086475	0.094618569
HALLMARK_INTERFERON_GAMMA_RESPONSE	0.352468193	1.585240551	0.01174743	0.094618569
HALLMARK_UV_RESPONSE_UP	0.406862224	1.63919149	0.012841091	0.094618569
HALLMARK_APICAL_JUNCTION	−0.386203056	−1.685607276	0.015831135	0.097208721

**Figure 4:**
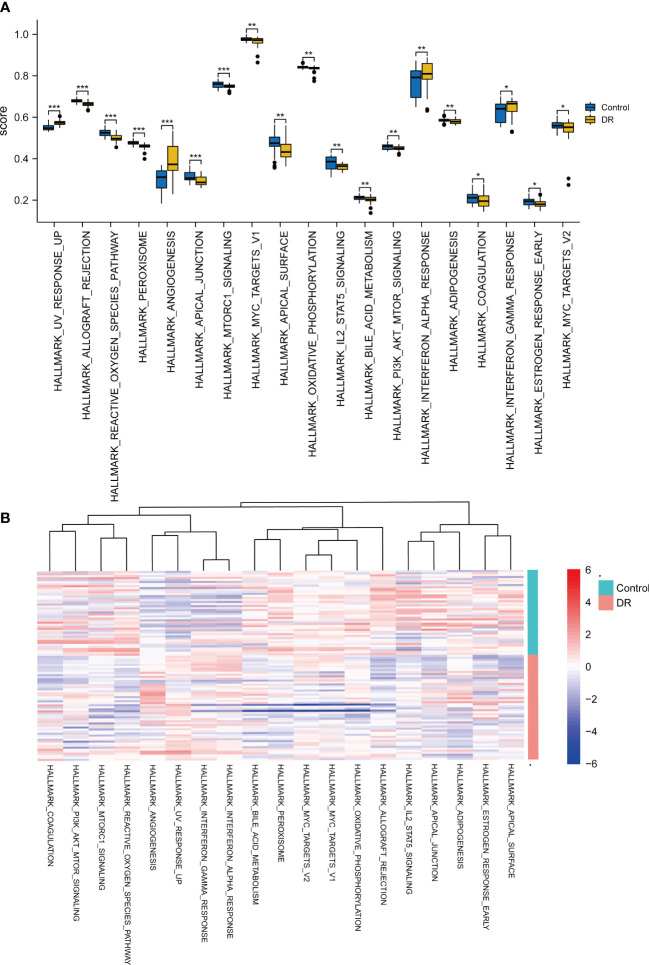
GSVA for dataset GSE146615. **(A)** Comparison plot of gene sets showing differences between diabetic retinopathy (DR) and control groups (^*^ *p* < 0.05; ^**^ *p* < 0.01; ^***^ *p* < 0.001). **(B)** Complex heatmap of gene sets showing differences between DR and control samples.

To examine the difference in hallmark gene sets between the DR samples and control samples, GSVA in the dataset GSE146615 was implemented. The group comparison plot (**Figure 4A**) and heat map (**Figure 4B**) displayed that a total of 19 hallmark gene sets exhibited marked differences between the DR and control samples (*p* < 0.05). There were extremely statistically significant differences in gene sets for UV response up, allograft rejection, the reactive oxygen species pathway, peroxisome, angiogenesis, apical junction, and mTORC1 signaling. Highly statistically significant gene sets were revealed in myelocytomatosis (MYC) target v1, apical surface, oxidative, phosphorylation, IL-2-STAT5 signaling, bile acid metabolism, PI3K/AKT/mTOR signaling, NF-α response, and adipogenesis. Statistically significant differences were also shown in coagulation, INF-γ response, estrogen response early, MYC targets V2.

### PPI and interaction analysis of miRNA targets with FRDEGs

**Figure 5:**
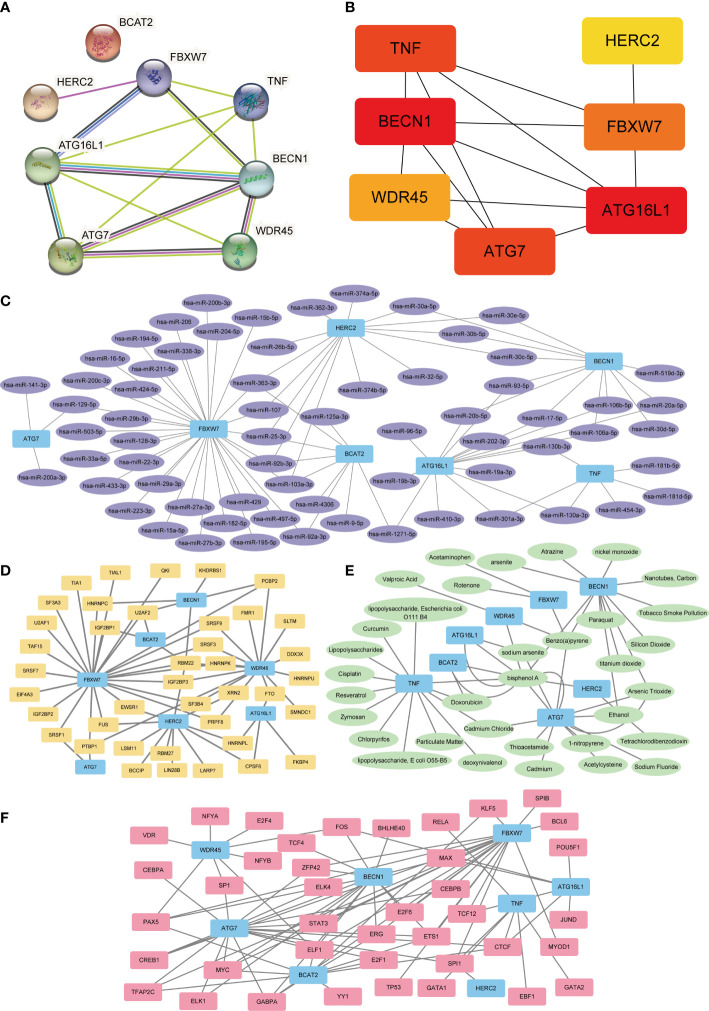
PPI, mRNA–miRNA, mRNA–TF, mRNA–drugs, and mRNA–RBP interaction networks. **(A)** PPI Network of FRDEGs. **(B)** Correlation interaction network diagram of FRDEGs in MCC algorithm. The block color from yellow to red indicates a gradual increase in rating. **(C)** mRNA-miRNA interaction networks predicted by FRDEGs. Blue represents mRNA molecules and purple represents miRNA. **(D)** FRDEGs interact with transcription factor (TF) in mRNA–TF network. Blue represents the mRNA molecule and yellow represents TF. **(E)** mRNA–drugs interaction network. Blue represents mRNA and green represents drugs**. (F)** mRNA–RBP interaction network. Blue represents mRNA and pink represents RBP. PPI, protein–protein interaction; FRDEGs, ferritinophagy-related differentially expressed genes; DR, diabetic retinopathy; TF, transcription factors; RBP, RNA-binding protein; MCC, maximum cross correlation.

A PPI network was constructed by introducing eight FRDEGs (HERC2, ATG16L1, WDR45, BCAT2, FBXW7, BECN1, TNF, ATG7) into STRING database (**Figure 5A**). BCAT2 was neither associated with other molecules and nor developed a molecular network. Then, seven other genes (HERC2, ATG16L1, WDR45, FBXW7, BECN1, TNF, ATG7) were ranked according to scores (Matthews Correlation Coefficient algorithm (MCC)) and were selected as hub genes for DR (**Table 3**; **Figure 5B**). The color of the blocks in the figure from yellow to red represents a gradual increase in the score.

**Table 3:** Top seven hub genes in network ranked by MCC score.

Rank	Gene ID	Gene name	MCC score	Change
1	BECN1	Beclin 1	18	Up
2	ATG16L1	Autophagy-related 16 like 1	18	Up
3	TNF	Tumor necrosis factor	12	Down
4	ATG7	Autophagy-related 7	12	Down
5	FBXW7	F-box and WD repeat domain containing 7	7	Up
6	WDR45	WD repeat domain 45	6	Up
7	HERC2	HECT and RLD domain-containing E3 ubiquitin protein ligase 2	1	Up

After predicting mRNA–miRNA interaction pairs, 91 miRNAs for eight FRDEGs were identified (**Figure 5C**). Interaction networks analysis of mRNA-TF showed 85 TFs combined with FRDEGs (**Figure 5D**). Furthermore, mRNA–drugs network analysis displayed 52 direct and indirect drug targets of 8 FRDEGs (**Figure 5E**). Finally, 64 RBPs were disclosed by mRNA-RBP interaction networks analysis (**Figure 5F**).

### Dataset validation of FRDEGs

**Figure 6:**
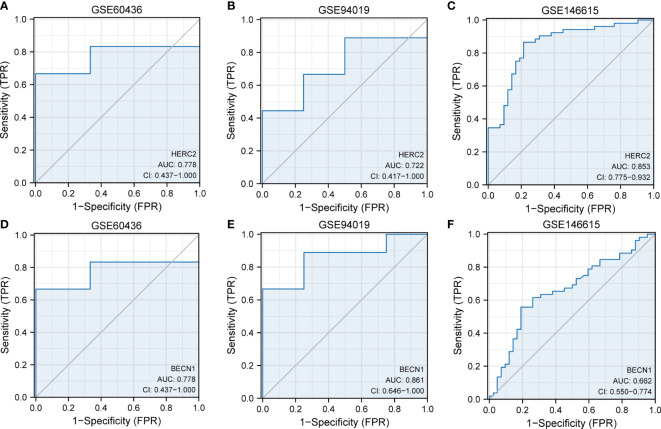
Dataset verification of FRDEGs. ROC curves of HERC2 gene in the datasets of **(A)** GSE60436, **(B)** GSE94019, **(C)** GSE146615, **(D)** GSE60436, **(E)** GSE94019, and **(F)** GSE146615. FRDEGs, ferritinophagy-related differentially expressed genes.

The GSE60436 and GSE94019 datasets were adopted to verify the eight FRDEGs. The outcomes of ROC curve monofactor analysis displayed that the diagnostic accuracies of the *HERC2* gene for DR was 77.8%, 77.2%, and 85.3% in the GSE60436, GSE 94019 and GSE146615 datasets, respectively (**Figures 6A-C**). The diagnostic accuracies of BECN1 gene were 77.8% in the GSE60436 dataset, 86.1% in the GSE 94019 dataset, but only 66.2% in the GSE146615 dataset (**Figures 6D-F**). These results showed that the genes HERC2 and BECN1 have potential for the diagnosis of DR.

### Associated matrix of immune cell infiltration

**Figure 7:**
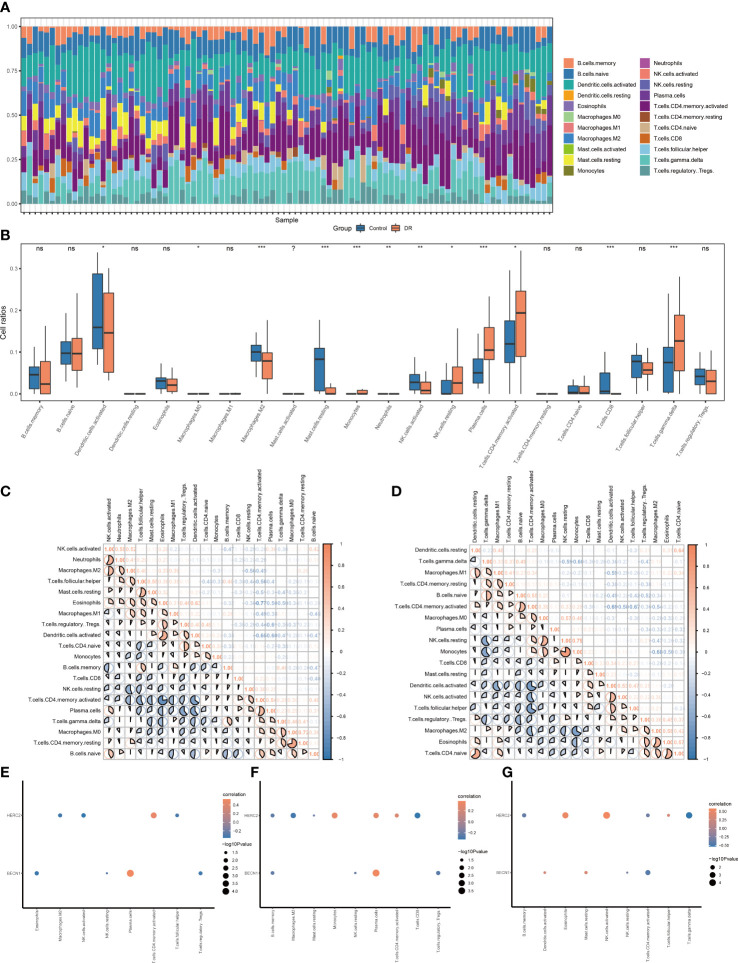
Immune infiltration landscape of the DR group and control group in dataset GSE146615. **(A)** Bar graphs of 22 immune cells. **(B)** Box plots of immune infiltration differences (ns, nonsignificant; ^*^ *p* < 0.05; ^**^ *p* < 0.01; ^***^ *p* < < 0.001, respectively). **(C, D)** Heat map of correlations of different immune cells in the **(C)** DR group and **(D)** control group, with red denoting positive correlation, blue representing negative correlation, and color intensity denoting degree of correlation. **(E–G)** Bubble plot of association of immune cell subsets with gene HERC2/BECN1 in the **(E)** DR group samples, **(F)** entire enrolled samples, and **(G)** control group samples. DR, diabetic retinopathy.

The correlations between 22 immune cells and eligible samples in the GSE146615 dataset were analyzed by means of CIBERSORT algorithm. The compositions of 22 immune cells in the samples were shown in a bar graph (**Figure 7A**). The two groups exhibited some differences in terms of immune infiltration (**Figure 7B**). The DR sample exhibited lower ratios of CD8+T cells, resting mast cells, M2 macrophages, activated NK cells, activated dendritic cells, and higher ratios of monocytes, plasma cells, activated CD4+T cells, resting NK cells, and gmma-delta (γδ) T cells. Next, the correlations of 22 immune cells were evaluated separately in DR group samples (**Figure 7C**) and control group samples (**Figure 7D**). There were negative correlations between most immune cell subsets in the two groups.

Specifically, we calculated the association of immune cell subsets with the gene HERC2/BECN1. In the 52 DR samples (**Figure 7E**), HERC2 had a negative association with follicular helper T cells, activated NK cells, and M2 macrophages and a positive association with activated CD4+T cells. BECN1 had negative association with eosinophils, Tregs, resting and NK cells, and had positive association with plasma cells. In total 94 DR and control samples (**Figure 7F**), HERC2 had negative association with memory B cells, M2 macrophages, resting mast cells, and CD8+T cells and a positive association with monocytes, plasma cells, and activated CD4+T cells. BECN1 had a negative association with memory B cells, resting NK cells, and Tregs and a positive association with plasma cells. In the 42 normal control samples (**Figure 7G**), HERC2 had negative association with γδ T cells, activated CD4+T cells, and memory B cells and a positive association with eosinophils, follicular helper T cells, and activated NK cells. BECN1 had negative association with activated CD4+T cells and resting NK cells and a positive association with resting mast cells and activated dendritic cells.

### External verification of FRDEGs

**Figure 8:**
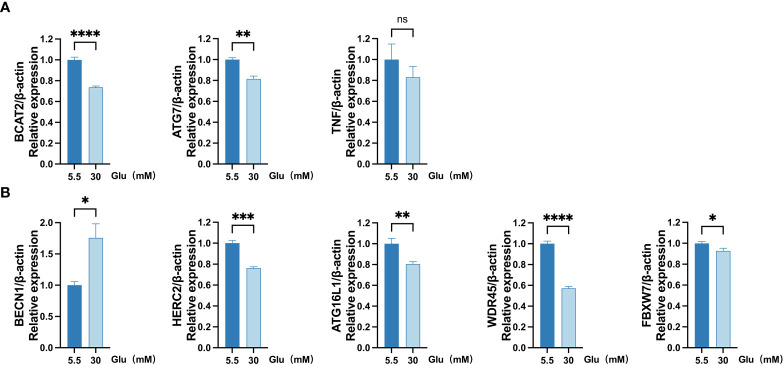
Measurement of mRNA levels of eight FRDEGs in ARPE-19 cells by qRT-PCR. **(A)** mRNA levels of BCAT2, TNF, and ATG7 were measured in cell samples. **(B)** mRNA levels of BECN1, HERC2, ATG16L1, WDR45, and FBXW7 were evaluated in cell samples. Two-sided unpaired Student’s *t*-test was conducted, with ns denoting nonsignificant; ^*^ *p* < 0.05; **p < 0.01; ***p < 0.001; ****p < 0.0001. FRDEGs, ferritinophagy-related differentially expressed genes; ARPE-19, retinal pigment epithelial cell line.

qRT-PCR analysis was implanted to validate the eight FRDEGs. The outcomes revealed that BECN1 expression was upregulated, while BCAT2 and ATG7 expressions were downregulated in ARPE-19 cells under the HG (30 mM glucose) environment for 48 h. The findings of the qRT-PCR analysis were in line with that of the bioinformatics analysis. Similar expected downregulated TNF levels were discovered in HG-treated group and the normal group. Levels of HERC2, ATG16L1, WDR45, and FBXW7, which should have been upregulated, were discovered to be decreased in HG-treated group (**Figure 8**).

## Discussion

DR is a diabetic complication with high specificity, complex multifactorial pathogenesis, and a devastating visual threat to the eyes (17). Numerous reports have displayed the involvements of oxidative stress (18), apoptosis, and autophagy (19) in DR development. However, there are disappointingly few treatment options for DR (20). Thus, a comprehensive understanding of the pathogenic therapeutic targets of DR is necessary.

Recent studies highlighted the function of ferritinophagy and ferroptosis in DR (16, 21–23). Ferritinophagy is a selective form of autophagy that is mediated by nuclear receptor coactivator 4 (NCOA4). It is crucially significant in the regulation of ferroptosis, mainly by regulating ROS production and intracellular iron homeostasis (12, 24). Therapeutic manipulation of ferritinophagy is a promising strategy for ferroptosis regulation in various metabolic stress settings (11). For instance, downregulation of ferritinophagy by ferritin heavy chain 1 (FTH-1) overexpression suppressed cell death induced by ferroptosis in the model of Parkinson’s disease (25). Moreover, it was pointed out that lipopolysaccharide increased NCOA4 expression and intracellular Fe^2+^ level but reduced ferritin level, ultimately inducing ferroptosis, and targeting ferroptosis induced by ferritinophagy might prevent sepsis-induced cardiac damage (26). Nevertheless, the underlying mechanisms and specific inhibition of ferritinophagy in DR are not yet fully unraveled. Clarifying the potential FRDEGs and their correlations with the physiological and pathological processes of DR might find new directions for the diagnosis, treatment, and prognosis of DR.

We identified eight potential FRDEGs between the DR and normal samples, including HERC2, ATG16L1, WDR45, BCAT2, FBXW7, BECN1, TNF, and ATG7. The current studies indicate that these genes could regulate ferritinophagy in liver fibrosis, neurodegeneration, cardiomyocyte disease, and tumors (27–30). GO and KEGG enrichment analyses displayed close association of the genes with autophagy of mitochondrion, mitochondrion disassembly, autophagosome assembly, and other signal pathways. GSEA revealed that these genes significantly enriched six gene sets, including INF-α response, UV response up, INF-γ response, apical junction, apical surface, and allograft rejection. In this study, HG-treated (30 mM glucose) ARPE-19 cells were utilized to testify the function of potential FRDEGs of DR *in vitro*. RPE cells have been extensively used in DR research. The outer blood-retinal barrier is primarily composed of RPE cells, which have a close association with the occurrence of macular edema. The proliferation and migration of RPE cells are crucial for forming fibrovascular membranes in DR (31). Moreover, recent evidence suggests that the photoreceptors and RPE cells contribute to retinal vascular alterations in the early stages of DR (32).

The genes BECN1 and HERC2 were validated in two datasets from intraocular fibrovascular membrane samples. It has been verified that BECN1, a core autophagy modulator, has a close correlation with autophagy in retinal ischemia, DR, and glaucoma (33–35). In hepatic stellate cells, the upregulation of ELAV-like RNA-binding protein 1 promoted the accumulations of ferritinophagy and autophagosome by binding to BECN1 mRNA (3′-untranalated region (3′-UTR)) and improving BECN1 mRNA stability (36). A recent study showed that poly(rC)-binding protein 1 repressed ferritinophagy-mediated ferroptosis through binding to CU-rich elements in 3′-UTRs of BECN1 mRNA in head and neck cancer (37). Likewise, our results highlighted that BECN1 is a potential target for ferritinophagy manipulation in DR. The HERC2 gene encodes an E3 ubiquitin ligase that participates in many cellular processes *via* ubiquitylation regulation of various protein substrates (38). In spite of the inconsistent results of HERC2 expression in qRT-PCR analysis with those of bioinformatics analysis, the role of HERC2 in DR deserves additional study. We speculated that gene expression might be altered by differences in cell types as well as culture conditions. Previous studies displayed that autophagic degradation of NCOA4 and HERC2-mediated ubiquitination could reduce ferritinophagy flux (39). Our enrichment analysis of molecular function also emphasized ubiquitin and ubiquitin-like protein ligase bindings.

ATG7 and BCAT2 genes are also potential biomarkers and therapeutic targets for DR. ATG7 and BCAT2 expression in qRT-PCR was consistent with the findings of bioinformatics analysis and earlier studies, although the two genes failed to be validated. The reason was speculated to be the limited sample size of the validation set. ATG7 is essential for autophagosome formation. Knockout or knockdown of ATG7 and ATG5 upregulated ferritin expression, decreased Fe^2+^ levels, and limited erastin-induced ferroptosis in fibroblasts and cancer cells (40). A recent proteomic study revealed that upregulation of ATG7 was accompanied by the upregulation of ferritinophagy-related proteins, such as LC3, ferritin (FTL, FTH1), and NCOA4 in an arsenic trioxide-treated neuroblastoma cell line (41). Based on these results and our bioinformatics analysis, we speculate that ATG7 is involved in ferritinophagy regulation of DR. Lately, BCAT2, an aminotransferase that mediates sulfur amino acid metabolism, has been identified as a specific inhibitor of ferroptosis. In hepatocellular carcinoma cells (HepG2), ferroptosis inducers (sulfasalazine, sorafenib, and erastin) activated ferritinophagy and increased cellular Fe^2+^ levels, consequently activating AMPK phosphorylation and suppressing nuclear translocation of SREBP1, then inhibiting BCAT2 transcription (28). In PPI analysis, BCAT2 was neither associated with other molecules nor developed a molecular network. Nevertheless, qRT-PCR analysis exhibited the results similar to the bioinformatics analysis. The role of BCAT2 in ferritinophagy of DR needs further exploration.

Accumulating evidence indicates that low-grade inflammation and persistent leukocyte activation play a vital role in pathogenesis and development of DR (42). Increased proportions of circulating neutrophils and decreased lymphocytes were considered associated with the presence and severity of DR (43, 44). Our previous study revealed lower ratios of M2 macrophages in diabetic rats and HG-treated microglia cell line (45). On the other hand, disturbances of iron homeostasis could affect the innate and adaptive immunity. For example, iron can regulate NK cell activity, neutrophil recruitment and inflammation, and macrophage polarization. Iron can also influence the differentiation and activation of Th1, Th2, Th17, and CTL, as well as antibody response in B cells (46, 47). In this way, the immune microenvironment in DR was analyzed by means of CIBERSORT. The results showed decreased proportions of CD8+T cells, M2 macrophages, resting mast cells, activated dendritic cells, and activated NK cells and increased ratios of monocytes, plasma cells, activated CD4+T cells, resting NK cells, and γδ T cells. Particularly, high HERC2 expression was linked to decreased proportions of M2 macrophages, activated NK cells and Th cells and increased proportions of activated CD4+T cells. High BECN1 expression was linked to decreased proportions of eosinophils, Tregs, resting NK cells, and increased proportions of plasma cells. Taken together, we hypothesize the involvements of genes BECN1 and HERC2 in chronic inflammatory and immune processes of DR. More correlative studies are warranted to clarify the interactions between ferritinophagy, inflammation, and immune responses in DR.

Few investigations have previously reported this ferritinophagy-related gene signature and targeted molecule in DR. Nevertheless, our study has several shortcomings. Firstly, the absence of disease stage and prognostic data limited further exploration of the impact of the detected genes on prognosis and disease recovery. We will supplement the samples and prognostic information of DR for further analysis in the follow-up study. Secondly, further experimental verifications and clinical studies are necessary to detect the diagnostic and therapeutic performance of these predicted target genes. In the next step, we will focus on clarifying the biological functions of genes as well as their pharmacological activities in the DR models *in vitro* and *in vivo*. A variety of experimental methods, such as Western blot, quantitative real-time PCR, and immunohistochemistry, will be used to deepen our understanding of the molecular mechanism of DR. In addition, flow cytometry can be used to measure the different populations of immune cells in different pathogenic stages.

## Conclusions

To summarize, bioinformatics analysis revealed the identified eight potential ferritinophagy-related genes in DR. BECN1 and HERC2 displayed high diagnostic accuracies in validation sets. *In vitro* experiments were preliminarily verified that BECN1, ATG7, and BCAT2 may impact the development and progression of DR through regulating ferritinophagy. Several immune cells related to ferritinophagy could play potential roles in DR. The research can provide novel insights into molecular pathogenesis, potential biomarkers, and therapeutic targets for ferritinophagy in DR.

## Data availability statement

The original contributions presented in the study are included in the article/**Supplementary Material**. Further inquiries can be directed to the corresponding authors.

## Author contributions

FY designed the study, collected and analyzed data. FY and CW wrote the initial manuscript. YS and TC verified the analysis of data. WZ and XD edited and provided comments to improve the manuscript. WK and LC designed the experiment. SY and PW revised the manuscripts. All authors contributed to the article and approved the final manuscript.

## Supplementary material

Supplementary Figure 1 Flow chart of data analysis. DEGs, differentially expressed genes; FRDEGs, ferritinophagy-related DEGs; GO, Gene Ontology; GSVA, Gene Set Variation Analysis; PPI, protein-protein interaction; KEGG, Kyoto Encyclopedia of Genes and Genomes; TF, transcription factors; GSEA, Gene Set Enrichment Analysis; RBP, RNA binding protein; ROC, receiver operating characteristic.

Supplementary Figure 2
**(A)** The boxplot of dataset GSE146615 before standardized, with blue representing control group and yellow representing DR group. **(B)** The boxplot of dataset GSE146615 after standardized, with blue representing control group and yellow representing DR group.
