# Supplementary material for: Comprehensive analysis of ferritinophagy-related genes and immune infiltration landscape in diabetic retinopathy
Source: Front Endocrinol (Lausanne). 2023 Jul 14;14:1177488. doi: 10.3389/fendo.2023.1177488 (PMC10377661; doi:10.3389/fendo.2023.1177488)
Supplement: Supplementary file 2 [file Table_1.docx]

**Supplemental Table 1** Quantitative PCR primers utilized in the research

| Genes | Primer sequences (from 5' to 3') |
| --- | --- |
| β-actin | F: 5’-CATGTACGTTGCTATCCAGGC-3’ |
|  | R: 5’-CTCCTTAATGTCACGCACGAT-3’ |
| ATG7 | F: 5’-ATGATCCCTGTAACTTAGCCCA-3’ |
|  | R: 5’-CACGGAAGCAAACAACTTCAAC-3’ |
| ATG16L1 | F: 5’-AACCAAATGCAGCGGAAGGA-3’ |
|  | R: 5’-AAAGCTTAGTGCGCAGGTCT-3’ |
| BCAT2 | F: 5’-CCACCGTGTTAGTGCAACAG-3’ |
|  | R: 5’-GTTCATGGTTCCCACCTCGG-3’ |
| BECN1 | F: 5’-GAGGTTGAGAAAGGCGAGACA-3’ |
|  | R: 5’-AATTGTGAGGACACCCAAGCA-3’ |
| FBXW7 | F: 5’-CGACGCCGAATTACATCTGTC-3’ |
|  | R: 5’-CGTTGAAACTGGGGTTCTATCA-3’ |
| WDR45 | F: 5’-TGTGTACTCCTTCCCCGACA-3’ |
|  | R: 5’-TCCACAAGTTGCAGACTCCC-3’ |
| TNF | F: 5’-CTCGAACCCCGAGTGACAAG-3’ |
|  | R: 5’-TATCTCTCAGCTCCACGCCA-3’ |
| HERC2 | F: 5’-GCGCTGTCTTTTGCCTTTG-3’ |
|  | R: 5’-AGGAACCTGGTCGCTCTCTC-3’ |
