# Supplementary figures and images for: Comprehensive analysis of ferritinophagy-related genes and immune infiltration landscape in diabetic retinopathy
Source: Front Endocrinol (Lausanne). 2023 Jul 14;14:1177488. doi: 10.3389/fendo.2023.1177488 (PMC10377661; doi:10.3389/fendo.2023.1177488)

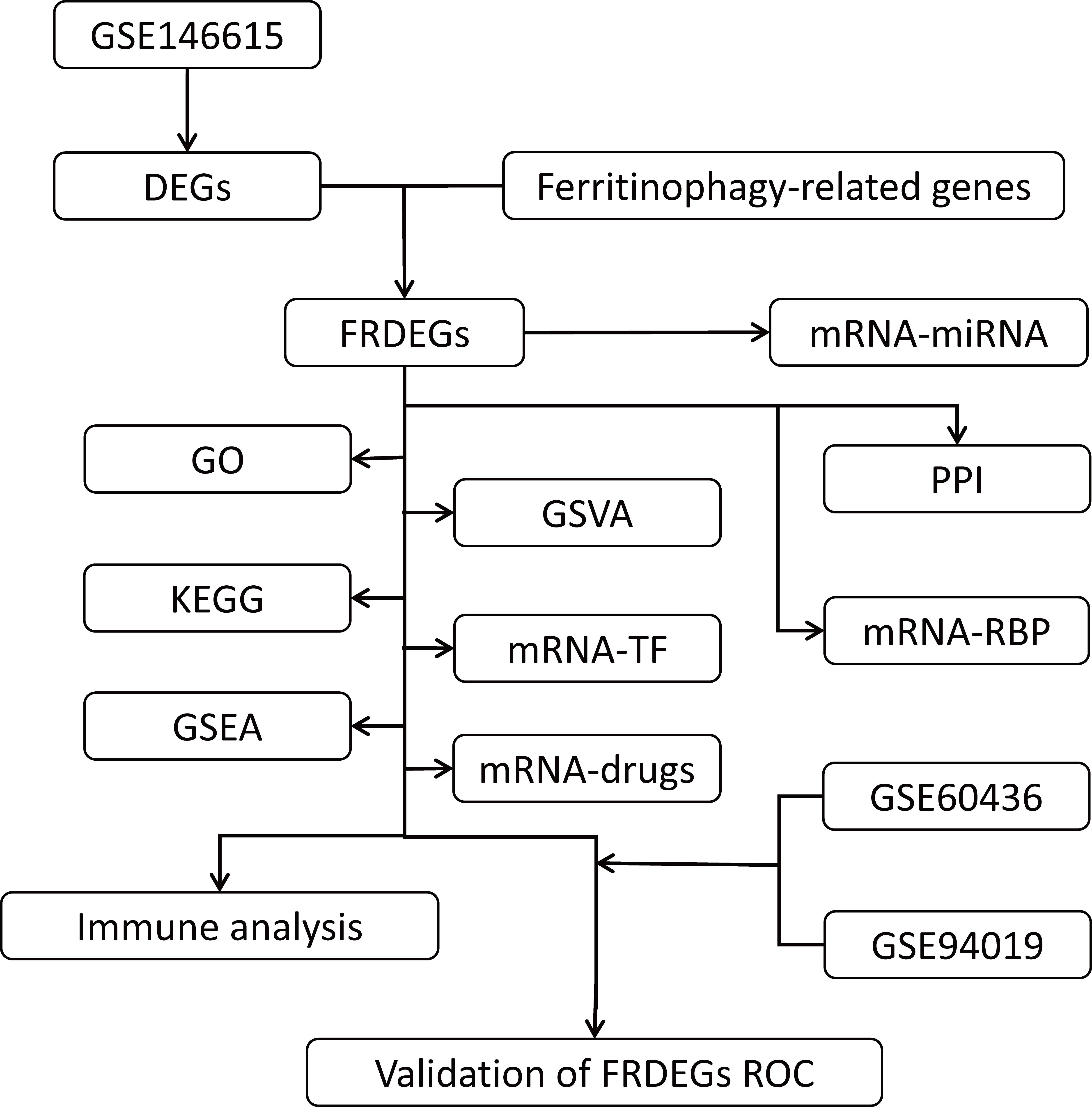

Supplement: Supplementary Figure 1 — Flow chart of data analysis. DEGs, differentially expressed genes; FRDEGs, ferritinophagy-related DEGs; GO, Gene Ontology; GSVA, Gene Set Variation Analysis; PPI, protein-protein interaction; KEGG, Kyoto Encyclopedia of Genes and Genomes; TF, transcription factors; GSEA, Gene Set Enrichment Analysis; RBP, RNA binding protein; ROC, receiver operating characteristic. [file Image_1.tif]

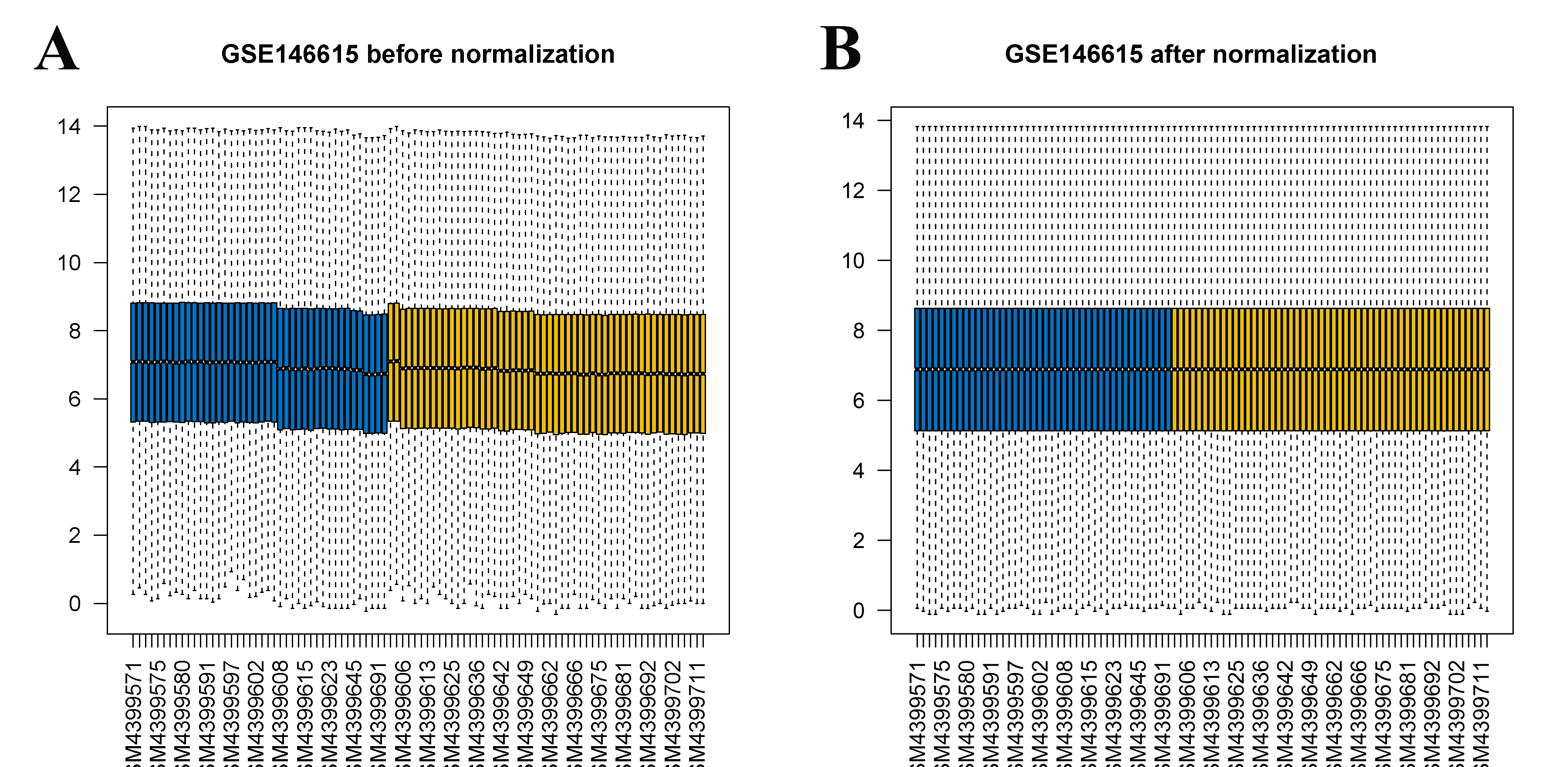

Supplement: Supplementary Figure 2 — (A) The boxplot of dataset GSE146615 before standardized, with blue representing control group and yellow representing DR group. (B) The boxplot of dataset GSE146615 after standardized, with blue representing control group and yellow representing DR group. [file Image_2.tif]
